# Absolute versus relative socioeconomic disadvantage and homicide: a spatial ecological case–control study of US zip codes

**DOI:** 10.1186/s40621-022-00371-z

**Published:** 2022-02-25

**Authors:** Ariana N. Gobaud, Christina A. Mehranbod, Beidi Dong, James Dodington, Christopher N. Morrison

**Affiliations:** 1 Department of Epidemiology, Mailman School of Public Health, Columbia University, 722 West 168th Street, Rm 516, New York, NY 10032 USA; 2 Department of Criminology, Law and Society, George Mason University, Fairfax, VA USA; 3 Department of Pediatrics and Emergency Medicine, Yale School of Medicine, New Haven, CT USA; 4 Department of Epidemiology and Preventive Medicine, Monash University School of Public Health and Preventive Medicine, Melbourne, Australia

**Keywords:** Income, Income inequality, Homicide

## Abstract

**Background:**

Homicide is a major cause of death and contributes to health disparities in the United States. This burden overwhelmingly affects people from racial and ethnic minority populations as homicide occurs more often in neighborhoods with high proportions of racial and ethnic minority residents. Research has identified that environmental factors contribute to variation in homicide rates between neighborhoods; however, it is not clear why some neighborhoods with high concentrations of racial and ethnic minority residents have high homicide rates while neighborhoods with similar demographic compositions do not. The aim of this study was to assess whether relative socioeconomic disadvantage, (i.e., income inequality), or absolute socioeconomic disadvantage (i.e., income) measured at the ZIP code- and state-levels, is associated with high homicide rates in US ZIP codes, independent of racial and ethnic composition.

**Methods:**

This ecological case–control study compared median household income and income inequality in 250 ZIP codes with the highest homicide rate in our sample in 2017 (cases) to 250 ZIP codes that did not experience any homicide deaths in 2017 (controls). Cases were matched to controls 1:1 based on demographic composition. Variables were measured at both the ZIP code- and state-levels.

**Results:**

Lower median household income at the ZIP code-level contributed most substantially to the homicide rate. Income inequality at the state-level, however, was additionally significant when controlling for both ZIP code- and state-level factors.

**Conclusions:**

Area-based interventions that improve absolute measures of ZIP code socioeconomic disadvantage may reduce gaps in homicide rates.

**Supplementary Information:**

The online version contains supplementary material available at 10.1186/s40621-022-00371-z.

## Background

Homicide is a leading cause of mortality and contributes to heath disparities in the United States. A total of 19,141 people were homicide victims in 2019 (CDC 2021). Risks for homicide are greater for men (Zahn et al. 1999; Massey and McKean 1985), adolescents and young adults (Zahn et al. 1999), and Black and Hispanic people (Hawkins et al. 1999; Sampson 1997). Homicide is the leading cause of death for Black men aged ≤ 44 and is a major contributor to differences in life expectancy between White and Black men (CDC 2016). The disproportionate exposure to homicide among Black and Hispanic people adds to disparities in physical injury and long-term mental and physical health (Sheats et al. 2018). Witnessing or hearing about violence such as homicide in communities can increase the propensity for becoming a victim or perpetrator of violence in adolescence (Finkelhor 2009; Margolin and Gordis 2004) or adulthood (Menard et al. 2014). Children and adolescents are especially vulnerable to increased risk of lifelong mental and physical health problems as a result of exposure to homicide (Danese et al. 2009). Identifying conditions that contribute to the occurrence of homicide is an essential step towards developing effective preventive interventions that reduce the absolute health burden and socio-demographically structured health disparities.

Absolute socioeconomic disadvantage (i.e., poverty) has been the leading focus of studies of macro-level correlates of crime, especially violent crime (assault, homicide, rape, and robbery) since the late 1970s (William Alex Pridemore 2011). The revival of social disorganization theory has further emphasized absolute socioeconomic disadvantage and its effect on neighborhoods (Bellair and Browning 2010; Bursik 1988; Sampson et al. 1997). Social disorganization theory suggests that a person’s residential location is more significant than the person’s characteristics when predicting criminal activity. Moreover, poverty and the concentration of poor economic conditions lead to social disorganization through a breakdown of social cohesion and norms. The consequence is socially structured hardship that result in feelings of “resentment, frustration, hopelessness, and alienation” which the theory suggests leads to widespread social disorganization and violent crime (Blau and Blau 1982). In a meta-analysis, poverty was found to be in a group of the most consistent macro-level predictors of violent crime (Pratt and Cullen 2005).

Empirical studies generally support these theoretical predictions regarding geographic distributions of violent crime. Research identifies that violent crime, including homicide, concentrates in specific neighborhoods (Braga et al. 2010; Sherman et al. 1989). For example, from 1980 to 2008, 74% of crimes in Boston occurred in 5% of city blocks (Braga et al. 2010). In Seattle, crime reduction was due mostly to crime declines in a small group of street segments from 1989 to 2002 (Weisburd et al. 2004). More recently, researchers have demonstrated that the concentration of crime at particular places is stable over time (Braga et al. 2010; Weisburd et al. 2004). A wide range of physical and social environmental conditions are associated with violent crime incidence in small areas (Dahlberg and Krug 2002; Anderson 1998). Violence is higher in communities where there are limited economic opportunities; where there are high concentrations of poor and unemployed people; and where there is greater residential instability (Sampson et al. 2002). One study found remediation of abandoned buildings in Philadelphia significantly reduced firearm violence as did vacant lot remediation (Branas et al. 2016). A number of possible mechanisms could explain these associations, including that remediation of abandoned buildings and vacant lots eliminate out-of-sight staging or storage areas for illegal firearms until they are needed (Garvin et al. 2013), or that increased informal surveillance results in a decrease of crime. Importantly, these mechanisms operate concurrent to broader macrosocial forces that affect crime and violence, including gentrification, development, and other structural and commercial determinants of health.

Though absolute socioeconomic disadvantage consistently predicts crime at the macro-level (Pratt and Cullen 2005), research has also shown relative socioeconomic disadvantage (i.e., income inequality) measured at both the macro- and individual-level are associated with violent crime (Chamberlain and Hipp 2015; Kawachi et al. 1999; Yitzhaki 1979). Relative socioeconomic disadvantage can be defined as the feeling of having less income than those around you (Eibner and Evans 2005). This feeling of inequality can have various negative effects for individuals. Prior research has shown the well-being of others can cause frustration among those who perceive themselves as having less. At the macro level, studies have identified income inequality as one of the most reliable predictors of homicide (Krohn 1976; Messner 1982). One study which focused on the relationship between youth and income inequality found that youth living at lower socioeconomic status in more affluent communities had an increased risk of being involved in criminal activity than youth living in poverty in more impoverished neighborhoods (Roger Jarjoura and Triplett 1997).

Disentangling the contribution of income and income inequality from the multiple potential confounders, including the demographic composition of the resident population, is a methodologically complex problem. Further, a social ecological systems perspective—the dominant theoretical framework that guides much epidemiologic research in neighborhoods and health (Krieger 2001; Roux and Mair 2010)—suggests that determinants of homicide will also be multi-scale and dynamic, and will reflect fundamental macrosocial causes of structural disadvantage that increase risk for crime and violence. For example, neighborhoods with high rates of poverty and income inequality will be related to distal social and economic policies at county, state, and federal levels. In addition, the strength of state-level measures of poverty and income inequality may affect crime in local areas. The extent to which local and state-level measures of relative and absolute measures of socioeconomic disadvantage are associated with homicide is poorly understood. A quantification of this relationship can enhance our understanding of the broader associations between socioeconomic disadvantage and homicide, while providing estimates that can be used in future theoretical assessment and empirical investigations in which income or income inequality is treated as a covariate.

The aim of this study was to identify whether income or income inequality at the ZIP code- or state-level contribute to homicide in small areas, operationalized as US ZIP codes. A matched ecological case–control design allowed us to address problems related to various highly correlated confounders, and to examine why some ZIP codes with high concentrations of racial and ethnic minority residents have high homicide rates while other such ZIP codes do not experience homicide.

## Methods

### Study design

The unit of analysis for this ecological case–control study were 2017 ZIP codes. Though an imperfect proxy for neighborhoods, we chose ZIP codes in 2017 as the unit of analysis because of data availability. ZIP codes eligible for inclusion were in the 34 US states and four counties in California that participated in the National Violent Death Reporting System (NVDRS) during that year (n = 23,949). The Centers for Disease Control and Prevention (CDC) created the NVDRS in 2002 to collect data on all types of violent deaths—including homicides—in all settings for all age groups (State Profiles et al. 2019). The NVDRS has been fully described elsewhere (Paulozzi et al. 2004). Abstractors in participating states extract detailed information from the death certificate, coroner or medical examiner’s report, and police report to summarize violent deaths. Available data are victim demographic characteristics, weapons, suspects, victim–suspect relationships, location, and precipitating circumstances. The abstractor assigns a “type of death” code to the case and writes two brief narratives on each incident to summarize the coroner or medical examiner report and the police report.

We used the NVDRS to calculate counts of homicide for 2017 within eligible ZIP codes. Case units were the 250 ZIP codes that had the highest homicide rate and had ≥ 5 homicides in 2017 (to ensure the rate was stable). Controls were ZIP codes that had no homicides in 2017 (Rothman et al. 2008), frequency matched to cases at a ratio of 1:1. The matching procedure was performed using American Community Survey (ACS) 2013–2017 5-year estimates for ZIP Code Tabulation Areas (ZCTAs) (USA 2013–2017). We calculated a balanced matrix of the Euclidean distance in 6-dimensional space between all eligible ZIP codes based on 6 demographic characteristics identified in prior studies to be associated with increased homicide rates: proportion Black, proportion Hispanic, proportion Asian, proportion male, proportion aged 15–24, and proportion aged 25–34 (Beard et al. 2017; Wintemute 2015). Cases were matched to the eligible control that was closest in Euclidian distance, located in a different state, and contained the same USDA Rural–Urban Continuum Code classification (urban, micropolitan, small town, or rural). This procedure ensured that cases were the most demographically similar to their corresponding control from among all ZIP codes in the 34 states and four counties in California participating in the NVDRS in 2017, while allowing assessment of associations for both ZIP code-level and state-level exposures. The total analytic sample was 500 ZIP codes. The reason for this sample was a trade-off between feasibility and statistical power as there was an intensive data collection component.

### ZIP code-level measures

We measured two independent variables at the ZIP code-level differentiating between income and income inequality. We used ACS 2013–2017 5-year estimates to obtain median household income and the Gini coefficient for each ZIP code. The Gini coefficient, though documented with limitations, has become a standard measurement of income inequality (US Census Bureau 2013–2017). It ranges from zero, expressing perfect equality (where all persons have equal shared of aggregate income), to one, expressing maximal inequality (where one person has all the income and the rest have none). The measurement characterizes the distribution of income within a social unit or group of people and therefore has no individual level analogue.

Other independent measures at the ZIP code-level we controlled for included population size, population density, percent of the population that was unemployed, percent land use (proportion industrial, retail, and green space) (Morrison et al. 2019), and walkability. We measured population density as population per km^2^ using the ACS 2013–2017 5-year estimates (). We overlaid the ZIP codes on county parcel files we obtained from the US Census Bureau to calculate percent of land area that is retail, industrial, and green space (TIGER 2017). To assess walkability, we used the walk score provided by WalkScore™ (WalkScore 2020). Walk scores range from 0 to 100. Values closer to 0 signify car dependent ZIP codes and increasing values correspond to increasing walkability.

### State-level measures

We measured two independent variables at the state-level, once again differentiating between income and income inequality. We used ACS 2013–2017 5-year estimates to obtain median household income and the Gini coefficient for each state.

Other independent measures at the state-level we controlled for included percentage of the population who reported being Black, Asian, or Hispanic, and percentage of the population who were male (; McClenathan et al. 2019). We again used the ACS 2013–2017 5-year estimates for these data.

### Statistical analysis

We compared distributions of the variables between case and control ZIP codes using Students’ t-test and by visual inspection of scatter plots. We used multilevel logistic regression models to assess the odds that ZIP code-level or state-level median household income and the Gini coefficient are associated with homicide. Model 1 assessed the association at the ZIP code-level while controlling for ZIP code-level variables, model 2 the state-level while controlling for state-level variables, and model 3 combined both models 1 and 2. We examined the variance inflation factor (VIF) in each model to measure the amount of multicollinearity between variables. Although the matching procedure conditioned upon 6 key ZIP code-level demographic characteristics (proportion Black, proportion Hispanic, proportion Asian, proportion male, proportion aged 15 to 24, and proportion aged 25 to 34), there may be residual confounding by these characteristics (Pearce 2016). We controlled statistically for these 6 characteristics in all 3 models.

We conducted sensitivity analyses comparing the results of the matched case control study to a simple random sample of controls. To maintain comparability between the cases and controls, we set the minimum population size of an eligible control to 25,000. The population of the smallest case ZIP code was 26,500. We set a seed and used the random sample function in R to select our controls. We conducted all statistical analyses in R version 4.0.4.

## Results

### Descriptive statistics

**Fig. 1:**
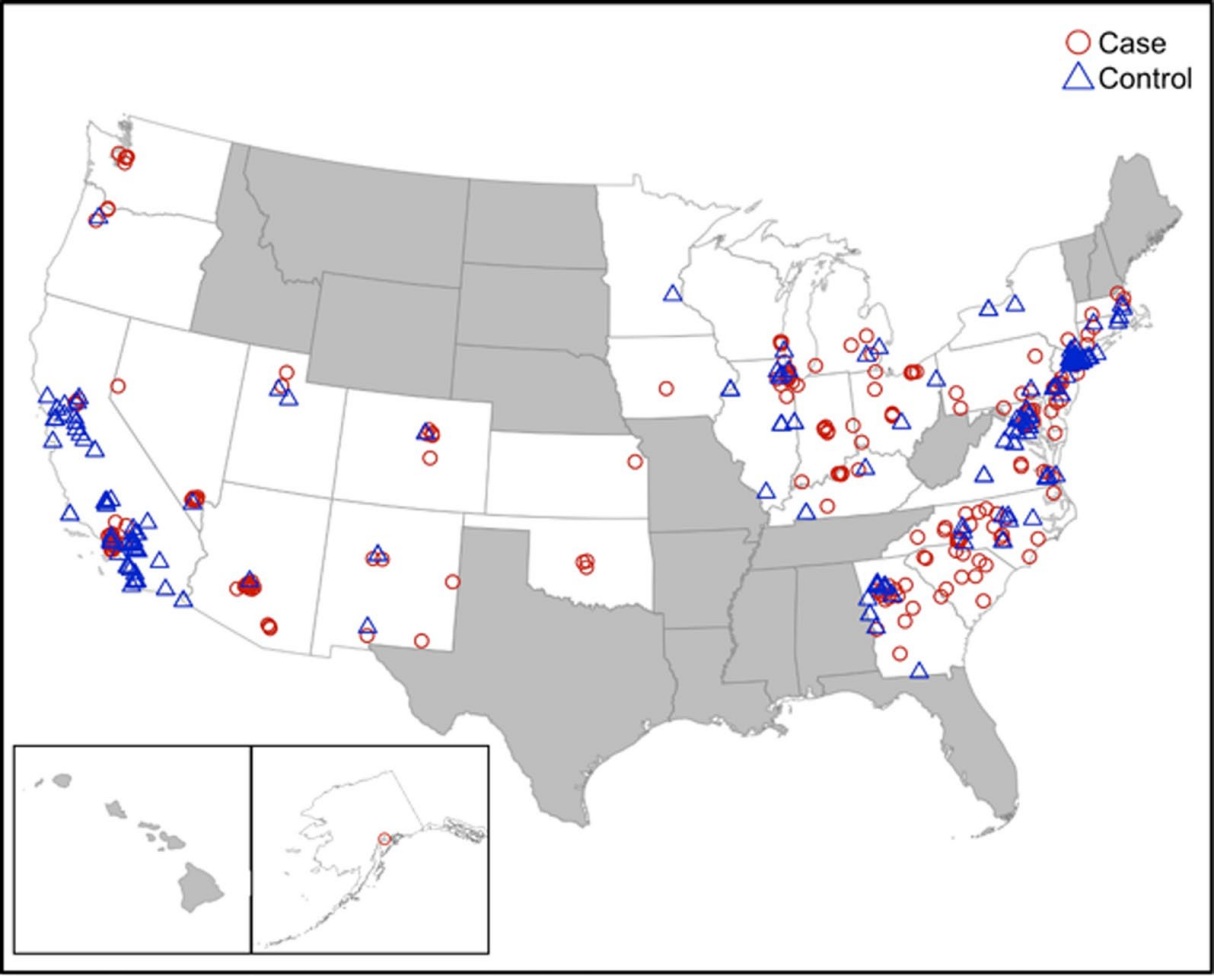
Matched cases and controls from participating NVDRS states in 2017. Cases and controls were selected from the 34 states and four counties in California participating in the CDC's National Violent Death Reporting System (NVDRS). Case units were defined as the 250 ZIP codes with the highest per capita incidence of violent homicide deaths in 2017. Selected cases had $\ge$ 5 deaths. ZIP codes eligible for selection as control units (1) had no violent deaths in 2017 and (2) were located within the 35 NVDRS states. Cases and controls were matched on proportion Black, proportion Hispanic, proportion Asian, proportion male, proportion aged 15–24, and proportion aged 25–34

Thirty-one of the 35 states in the NVDRS in 2017 were represented in our study (Fig. 1). The most case and control ZIP codes came from California (*n* = 40 and *n* = 62 respectively). Matched case and control ZIP codes were similar in the mean percent of the population aged 15–24, racial demographics, and percent male. They differed, however, in the mean population size and percent of the population 25–34 years old (Table 1). For example, a case ZIP code in New York was matched to a control ZIP code in California. In the case ZIP code, the population consisted of a mean of 13% aged 15–24, 17% aged 25–34, 35% Black, 6% Asian, 52% Hispanic, and 48% male. In the control ZIP code, the population consisted of a mean of 17% aged 15–24, 15% aged 25–34, 12% Black, 10% Asian, 58% Hispanic, and 50% male. In the same matched pair, the mean population in the case ZIP code was 102,718 compared to 94,327 in the control ZIP code. An additional file shows the results of the case–control matching in more detail (see Additional file 1).

**Table 1:** Distribution of ZIP code-level and state-level attributes for matched cases and controls^1^

	Cases (*n* = 250) Mean (SD)	Controls (*n* = 250) Mean (SD)	*P* value
*Zip code level*			
Income			
Median household income (USD)	46,342.39 (11,415.10)	63,917.91 (19,786.93)	** < 0.0001**
Income inequality			
Gini coefficient	0.45 (0.04)	0.43 (0.05)	** < 0.0001**
Population size	50,197.1 (18,438.84)	45,780.38 (15,963.55)	**0.0045**
Age group			
% 15–24	15.07 (4.95)	14.52 (5.18)	0.2301
% 25–34	15.70 (2.77)	14.94 (2.60)	**0.0016**
Race ethnicity			
% Black	28.43 (24.71)	25.94 (24.23)	0.2554
% Asian	4.47 (5.40)	4.94 (5.01)	0.3181
% Hispanic	30.26 (26.43)	28.95 (24.66)	0.5685
% Male	48.57 (2.17)	48.62 (1.78)	0.7881
% Unemployed	4.20 (1.28)	3.79 (1.18)	** < 0.0001**
Land use			
% Land area that is retail	2.03 (3.24)	4.82 (6.80)	** < 0.0001**
% Land area that is industrial	2.53 (4.57)	13.98 (52.00)	**0.0006**
% Land area that is greenspace	10.75 (14.71)	49.88 (70.82)	** < 0.0001**
Population density (per km^2^)	3424.29 (5440.35)	3400.32 (6592.38)	0.9647
Walk Score	42.31 (29.58)	35.34 (30.78)	**0.0101**
*State level*			
Income			
Median household income	60,182.41 (9297.23)	65,172.5 (8350.00)	** < 0.0001**
Income inequality			
Gini coefficient	0.47 (0.02)	0.48 (0.02)	** < 0.0001**
Race ethnicity			
% Black	13.30 (8.86)	14.82 (8.57)	**0.0512**
% Asian	6.00 (4.23)	8.13 (4.01)	** < 0.0001**
% Hispanic	18.13 (12.60)	20.64 (12.06)	**0.0233**
% Male	49.18 (0.57)	49.03 (0.51)	**0.0014**

^1^Cases and controls were selected from the 34 states and four counties in California participating in the CDC's National Violent Death Reporting System (NVDRS). Case units were defined as the 250 ZIP codes with the highest per capita incidence of violent homicide deaths in 2017. Selected cases had $\ge$ 5 deaths. ZIP codes eligible for selection as control units (1) had no violent deaths in 2017 and (2) were located within the 35 NVDRS states. Cases and controls were matched on proportion Black, proportion Hispanic, proportion Asian, proportion male, proportion aged 15–24, and proportion aged 25–34

*Bolded values are statistically significant at an alpha of 0.05

Case ZIP codes were more likely than controls to have lower median household income ($46,342 vs. $63,918 respectively) and greater income inequality (0.45 vs. 0.43 respectively) (Table 1). Case ZIP codes were also more walkable and had lower percentage of land that is retail and industrial and higher percentage of land that is greenspace when compared to the control ZIP codes (Table 1).

Case ZIP codes were in states with lower median household income ($60,182 vs. $65,173). Control ZIP codes tended to be in states with higher percentages of Black, Asian, and Hispanic populations (Table 1).

### Model results

There was no concern for multicollinearity in any of the models as the VIF for all variables was below 2. In model 1, when controlling for only ZIP code-level variables, a $10,000 increase in ZIP code-level median household income was associated with an 85% lower odds of being a case ZIP code (OR 0.15; 95% CI 0.08, 0.27) (Table 2). When controlling for only state-level variables in model 2, a $11,415.10 increase in state-level median household income was associated with a 46% lower odds of being a case ZIP code (OR 0.54; 95% CI 0.38, 0.78). Similarly, a one unit increase in the state-level Gini coefficient was associated with a 46% lower odds of being a case ZIP code (OR 0.54; 95% CI 0.36, 0.82 respectively). After controlling for both ZIP code- and state-level variables in model 3, a $11,415.10 increase in ZIP code-level median household income was associated with an 83% lower odds of being a case ZIP code and a one unit increase in the state-level Gini coefficient was associated with a 56% lower odds of being a case ZIP code (OR 0.17; 95% CI 0.09, 0.31 and OR 0.44; 95% CI 0.23, 0.83 respectively). An additional files shows sensitivity analyses using simple random sample of controls produced similar results (see Additional file 2).

**Table 2:** Odds ratios and 95% confidence intervals for homicide in matched cases and controls^1^

	Model 1			Model 2			Model 3		
	OR	95% CI		OR	95% CI		OR	95% CI	
*ZIP code-level*									
Median household income	**0.15**	**0.08**	**0.27**				**0.17**	**0.09**	**0.31**
Gini coefficient	1.03	0.70	1.52				1.35	0.92	1.98
*State-level*									
Median household income				**0.54**	**0.38**	**0.78**	1.15	0.66	2.02
Gini coefficient				**0.54**	**0.36**	**0.82**	**0.44**	**0.23**	**0.83**

^1^Cases and controls were selected from the 34 states and four counties in California participating in the CDC's National Violent Death Reporting System (NVDRS). Case units were defined as the 250 ZIP codes with the highest per capita incidence of violent homicide deaths in 2017. Selected cases had $\ge$ 5 deaths. ZIP codes eligible for selection as control units (1) had no violent deaths in 2017 and (2) were located within the 35 NVDRS states. Cases and controls were matched on proportion Black, proportion Hispanic, proportion Asian, proportion male, proportion aged 15 to 24, and proportion aged 25 to 34. All models controlled for matched variables. Model 1 adjusted for ZIP code-level variables. Model 2 adjusted for state-level variables. Model 3 adjusted for all variables

^*^Bolded values are statistically significant at an alpha of 0.05

## Discussion

This spatial ecological matched case–control study of US ZIP codes identified that income, not income inequality, at the local level is associated with homicide, independent of age, race/ethnicity, and sex. Specifically, lower median household income at the ZIP code-level contributed most substantially to homicide. Income inequality at the state-level, however, was additionally significant when controlling for both ZIP code- and state-level factors.

Our findings advance the collective understanding of violent crime in US ZIP codes with respect to the impacts of socioeconomic disadvantage. Research has identified that income inequality contributes to variation in homicide between neighborhoods, and these neighborhoods tend to have high proportions of racial and ethnic minority residents (Beard et al. 2017). Black and Hispanic persons in the US are more likely to live in poverty than white persons and more likely to encounter difficulties when improving their economic situations (Williams 1997). This increased risk has been attributed to economic inequality (Blau and Blau 1982). Previous work has found strong positive associations between income inequality and homicide rates. It has been suggested that connectedness and community-level collective efficacy are protective factors that may offset many of the negative influences of income inequality (Wilson and Daly 1997; Rowhani-Rahbar et al. 2019). However, results have been inconclusive about the influence of relative and absolute predictors of socioeconomic disadvantage at the local- or state-level. Our findings demonstrate that income at a local level has the greatest impact. This may be accounted for by the differences in how the two variables effect violent crime rates: income inequality, as a measure of *relative* socioeconomic disadvantage, captures the effect of the individual’s relationship to larger society, whereas poverty, a measure of *absolute* socioeconomic disadvantage, captures the effect of resource deprivation on individuals. This finding is consistent with theory that suggests at lower levels of aggregation, individual or absolute income will impact health more than income inequality (Wilkinson 1996). It is only within larger geographic areas that the social heterogeneity which is necessary for the effect of income inequality to occur that one finds a relationship between income inequality and health.

Our results concord with guiding theories of the etiology of violent crime. The routine activities theory holds that crime occurs with the convergence in space and time of motivated offenders, suitable targets, and the absence of capable guardianship (Cohen and Felson 1979). It follows that removing any one of these elements is a sufficient condition to prevent crime from occurring. For example, locating active or passive guardianship (e.g., police, security guards, door staff, friends, neighbors, CCTV cameras) at a location will reduce the occurrence of crime in that setting. Small area variation in population size, composition, and flow will alter the balance of offenders, targets, and guardians in ways that encourage or discourage crime (Cohen and Felson 1979). Increased poverty (i.e., greater absolute socioeconomic disadvantage) will furthermore increase the presence of motivated offenders, leading to greater crime incidence. Differences in physical conditions, such as poor street lighting, will have similar effects (Culyba et al. 2016). Further, social disorganization and collective efficacy suggest that formal and informal agents of social control—such as police presence and high social cohesion among neighbors—will deter violent crime due to increased risks that offenders will be detected and punished (Beccaria 1986). Several studies have observed that the concentration of potential offenders in neighborhood areas, measured by neighborhood economic socioeconomic disadvantage, is positively associated with crime rates (Andersen 2006; Miethe and McDowall 1993). Our findings might further identify that lower income at the ZIP code-level would give rise to an increased presence of motivated offenders. Additionally, guardianship of a place or geographic area is related to the presence of individuals or systems that can monitor or regulate behavior, whether it is formal (e.g. security guard or police) or informal (e.g. friends or neighbors) (Cohen and Felson 1979). For example, higher percentages of retail land area can increase the presence of guardians such as customers, and thus decrease the potential for crime.

This study should be interpreted with its limitations in mind. First, the NVDRS data for 2017 were available from a limited number of states and therefore are not nationally representative. Second, despite its universality and scalability, there are important limitations of the Gini coefficient. One drawback is the coefficient does not take into consideration structural changes in a population. Such changes can significantly influence the economic inequality in a population and complicate the comparison of coefficients between groups. Third, it included only associative analysis and cannot suggest causative mechanisms by which disparities in homicide develop and persist. However, these analyses make several critical contributions. By matching on age, race/ethnicity, and sex and controlling for them in the analysis, we eliminated any potential bias we may have introduced through matching and isolated ZIP code-level associations with homicide (Pearce 2016). Additionally, the matched approach yielded a more statistically efficient way to deal with confounding compared to the simple random sample selection of controls.

## Conclusions

Multi-level studies of income and income inequality are important to understand the characteristics, independent of race and ethnicity, that contribute to increased homicide rates. Our findings demonstrate that ZIP code-level income and state-level income inequality are associated with high homicide rates in ZIP codes that are otherwise demographically similar. It is important to understand these findings in the context of macrosocial determinants of health which are difficult to shift and will require a concerted effort over decades. Public–private partnerships are likely needed to address large infrastructure and economic drivers of violence. Moreover, alleviating low income in local areas and income inequality over larger areas could help reduce homicide rates. Far more research is needed as well as coordinated efforts to establish partnerships to impact upstream divers of poverty.

## Supplementary Information

**Additional file 1. **Title of data: Matched cases and controls from participating NVDRS states in 2017. Description of data: Cases and controls were selected from the 34 states and four counties in California participating in the CDC's National Violent Death Reporting System (NVDRS). Case units were defined as the 250 ZIP codes with the highest per capita incidence of violent homicide deaths in 2017. Selected cases had ≥ 5 deaths. ZIP codes eligible for selection as control units (i) had no violent deaths in 2017 and (ii) were located within the 35 NVDRS states. Cases and controls were matched on proportion Black, proportion Hispanic, proportion Asian, proportion male, proportion aged 15 to 24, and proportion aged 25 to 34.

**Additional file 2. **Title of data: Randomly selected cases and controls from participating NVDRS states in 2017. Description of data: Cases and controls were selected from the 34 states and four counties in California participating in the CDC's National Violent Death Reporting System (NVDRS). Case units were defined as the 250 ZIP codes with the highest per capita incidence of violent homicide deaths in 2017. Selected cases had ≥ 5 deaths. ZIP codes eligible for selection as control units (i) had no violent deaths in 2017 and (ii) were located within the 35 NVDRS states. Eligible controls had a population ≥ 25,000 and were randomly selected with replacement.

## Data Availability

Data from the National Violent Death Reporting System can be accessed upon request from the CDC. All other data is publicly available from the Census Bureau and WalkScore’s website.

## Abbreviations

NVDRS: National Violent Death Reporting System
US: United States
ACS: American Community Survey
ZCTA: ZIP Code Tabulation Areas
CDC: Centers for Disease Control and Prevention
