# Supplementary material for: Absolute versus relative socioeconomic disadvantage and homicide: a spatial ecological case–control study of US zip codes
Source: Inj Epidemiol. 2022 Feb 25;9:7. doi: 10.1186/s40621-022-00371-z (PMC8876118; doi:10.1186/s40621-022-00371-z)
Supplement: Supplementary file 1 — Additional file 1. Title of data: Matched cases and controls from participating NVDRS states in 2017. Description of data: Cases and controls were selected from the 34 states and four counties in California participating in the CDC's National Violent Death Reporting System (NVDRS). Case units were defined as the 250 ZIP codes with the highest per capita incidence of violent homicide deaths in 2017. Selected cases had ≥ 5 deaths. ZIP codes eligible for selection as control units (i) had no violent deaths in 2017 and (ii) were located within the 35 NVDRS states. Cases and controls were matched on proportion Black, proportion Hispanic, proportion Asian, proportion male, proportion aged 15 to 24, and proportion aged 25 to 34. [file 40621_2022_371_MOESM1_ESM.docx]

**
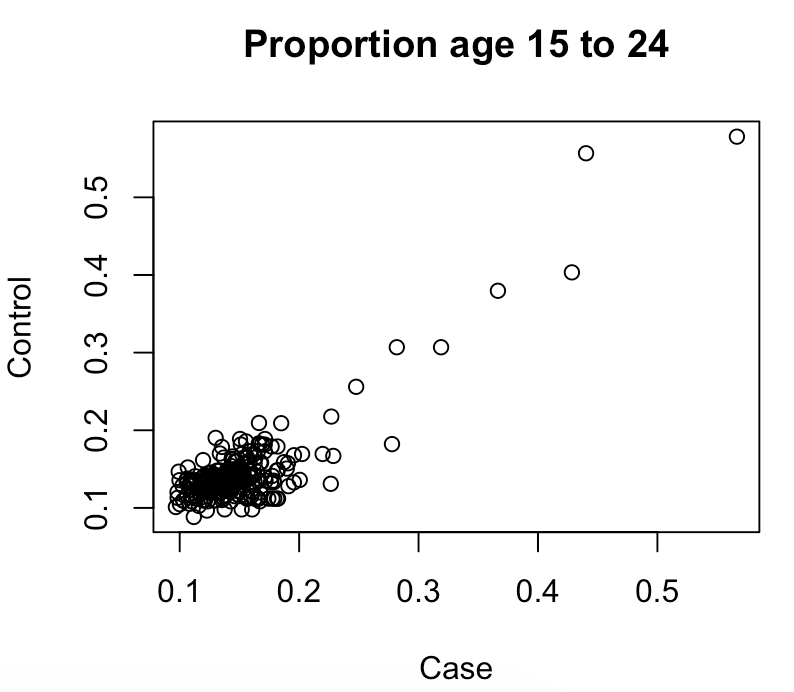

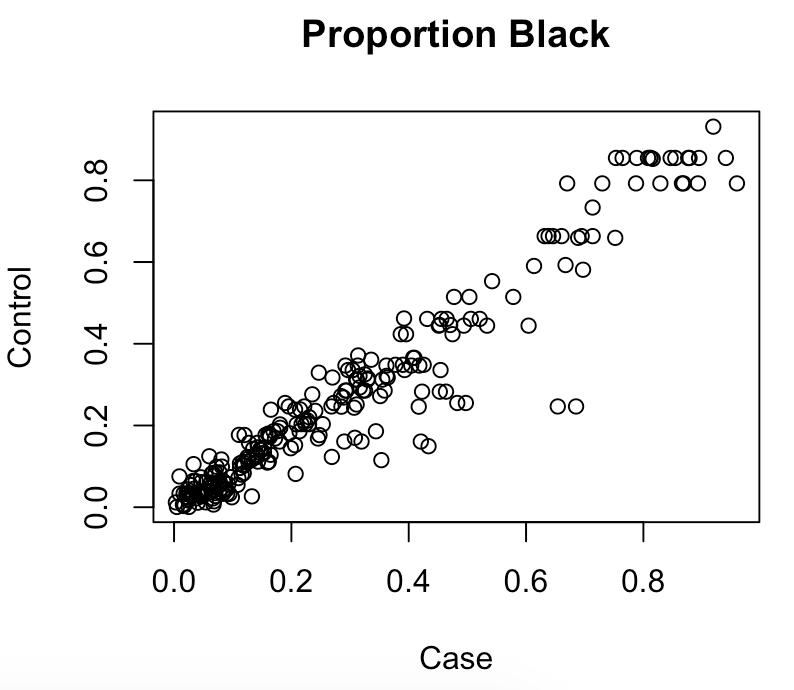
**

**
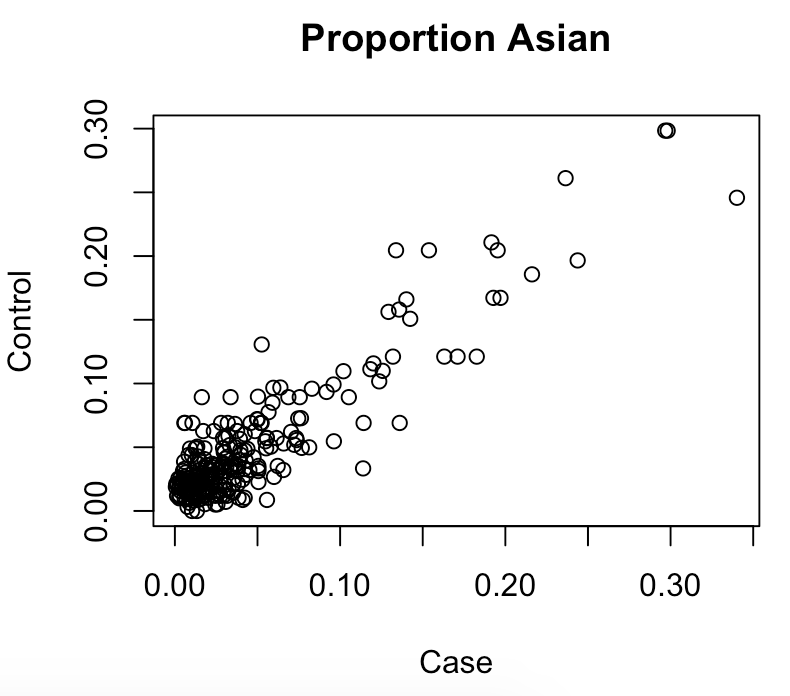

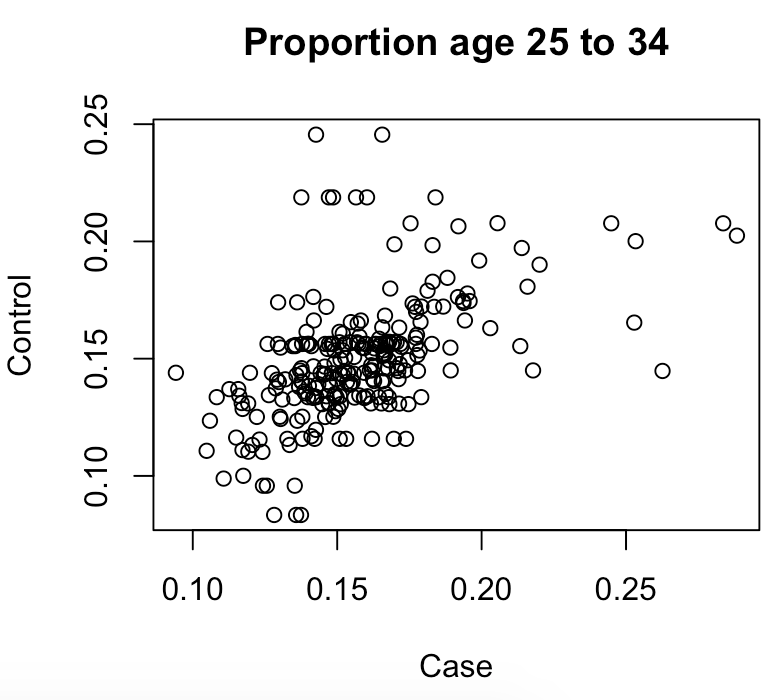
**

**
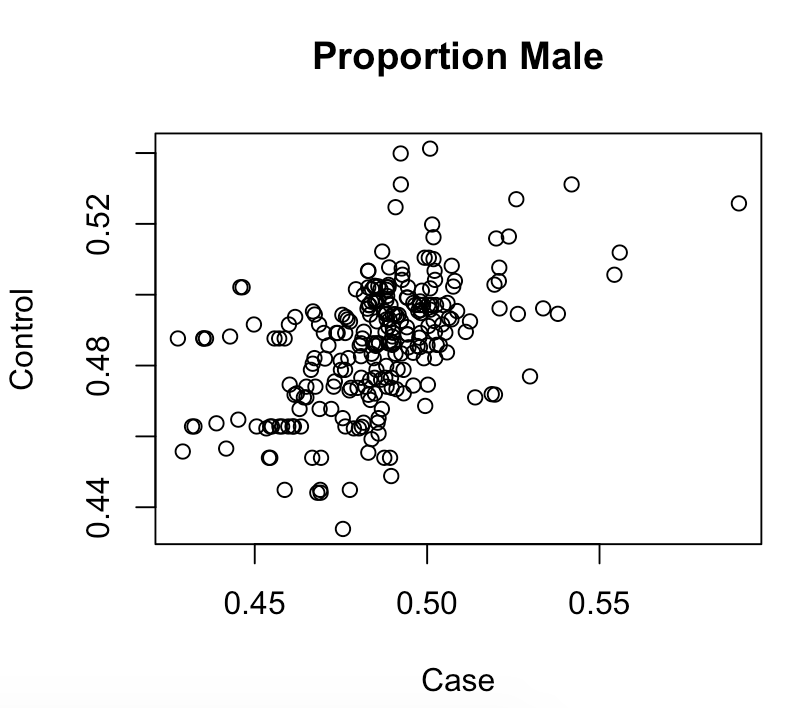

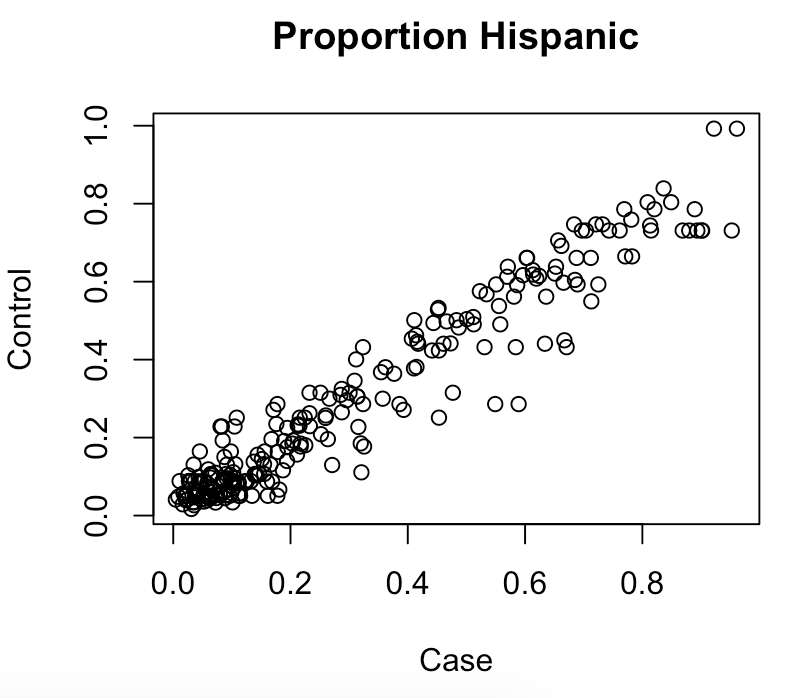
**

**Figure 1**. Plots of case-control matched variables

Results from a balanced matrix of the Euclidean distance in 6-dimensional space between all eligible ZIP codes based on 6 demographic characteristics identified in prior studies to be associated with increased incidence of violent homicide death: proportion Black, proportion Hispanic, proportion Asian, proportion male, proportion aged 15 to 24, and proportion aged 25 to 34. Cases were matched to the eligible control that was closest in Euclidian distance, located in a different state, and contained the same USDA Rural-Urban Continuum Code classification (urban, micropolitan, small town, or rural).
