# Supplementary material for: Absolute versus relative socioeconomic disadvantage and homicide: a spatial ecological case–control study of US zip codes
Source: Inj Epidemiol. 2022 Feb 25;9:7. doi: 10.1186/s40621-022-00371-z (PMC8876118; doi:10.1186/s40621-022-00371-z)
Supplement: Supplementary file 2 — Additional file 2. Title of data: Randomly selected cases and controls from participating NVDRS states in 2017. Description of data: Cases and controls were selected from the 34 states and four counties in California participating in the CDC's National Violent Death Reporting System (NVDRS). Case units were defined as the 250 ZIP codes with the highest per capita incidence of violent homicide deaths in 2017. Selected cases had ≥ 5 deaths. ZIP codes eligible for selection as control units (i) had no violent deaths in 2017 and (ii) were located within the 35 NVDRS states. Eligible controls had a population ≥ 25,000 and were randomly selected with replacement. [file 40621_2022_371_MOESM2_ESM.docx]

| **Table 1.** Distribution of ZIP code-level and state-level attributes for selected cases and randomly selected controls^1^ | | | | | | | | |
| --- | --- | --- | --- | --- | --- | --- | --- | --- |
|  |  | **Cases** (n = 250) Mean (SD) | |  | **Controls** (n = 250) Mean (SD) | |  | P Value |
| **Zip Code Level** | |  |  |  |  |  |  |  |
| *Income* | |  |  |  |  |  |  |  |
|  | Median Household Income | 46342.39 | (11415.10) | | 76399.88 | (25151.21) | | **<0.0001** |
| *Income Inequality* | |  |  |  |  |  |  |  |
|  | GINI Coefficient | 0.45 | (0.04) |  | 0.43 | (0.05) |  | **0.0004** |
| Population size | | 50187.10 | (18438.84) | | 37688.38 | (12265.81) | | **<0.0001** |
| *Age Group* | |  |  |  |  |  |  |  |
|  | % 15 - 24 | 15.10 | (4.95) |  | 13.90 | (7.32) |  | 0.0380 |
|  | % 25 - 34 | 15.70 | (2.77) |  | 13.74 | (4.31) |  | **<0.0001** |
| *Race Ethnicity* | |  |  |  |  |  |  |  |
|  | % Black | 28.43 | (24.71) |  | 5.79 | (8.13) |  | **<0.0001** |
|  | % Asian | 4.47 | (5.40) |  | 8.39 | (10.19) |  | **<0.0001** |
|  | % Hispanic | 30.26 | (26.43) |  | 17.04 | (17.17) |  | **<0.0001** |
| % Male | | 48.57 | (2.17) |  | 49.12 | (1.43) |  | **0.001** |
| % Unemployed | | 4.20 | (1.28) |  | 2.82 | (1.04) |  | **<0.0001** |
| *Land Use* | |  |  |  |  |  |  |  |
|  | % Land Area that is Retail | 2.50 | (3.50) |  | 2.03 | (2.74) |  | 0.5923 |
|  | % Land Area that is Industrial | 3.32 | (4.73) |  | 1.51 | (2.85) |  | **0.0019** |
|  | % Land Area that is Greenspace | 9.51 | (13.21) |  | 14.24 | (15.99) |  | **0.0003** |
| Population Density (per km2) | | 3424.29 | (5440.35) |  | 1514.68 | (3424.31) | | **<0.0001** |
| Walk Score | | 42.31 | (29.58) |  | 31.60 | (30.37) |  | **<0.0001** |
|  |  |  |  |  |  |  |  |  |
| **State Level** | |  |  |  |  |  |  |  |
| *Income* | |  |  |  |  |  |  |  |
|  | Median Household Income | 60182.41 | (9297.23) |  | 63017.74 | (7530.50) | | **<0.0001** |
| *Income Inequality* | |  |  |  |  |  |  |  |
|  | GINI Coefficient | 0.47 | (0.02) |  | 0.48 | (0.02) |  | 0.2524 |
| *Race Ethnicity* | |  |  |  |  |  |  |  |
|  | % Black | 13.30 | (8.86) |  | 9.46 | (6.22) |  | **<0.0001** |
|  | % Asian | 6.00 | (4.23) |  | 7.70 | (4.84) |  | **<0.0001** |
|  | % Hispanic | 18.13 | (12.60) |  | 21.09 | (13.45) |  | **0.0115** |
| % Male | | 49.18 | (0.57) |  | 49.34 | (0.51) |  | **0.0012** |
| * Bolded values are statistically significant at an alpha of 0.05 | | | | | | | | |
| ^1^ Cases and controls were selected from the 34 states and four counties in California participating in the CDC's National Violent Death Reporting System (NVDRS). Case units were defined as the 250 ZIP codes with the highest per capita incidence of violent homicide deaths in 2017. Selected cases had $\geq$ 5 deaths. ZIP codes eligible for selection as control units (i) had no violent deaths in 2017 and (ii) were located within the 35 NVDRS states. Cases and controls were matched on proportion Black, proportion Hispanic, proportion Asian, proportion male, proportion aged 15 to 24, and proportion aged 25 to 34 | | | | | | | | |

| **Table 2**. Odds ratios and 95% confidence intervals for violent homicide in selected cases and randomly selected controls^1^ | | | | | | | | | | | | |
| --- | --- | --- | --- | --- | --- | --- | --- | --- | --- | --- | --- | --- |
|  |  | **Model 1** | | |  | **Model 2** | | |  | **Model 3** | | |
|  |  | OR | 95% CI | |  | OR | 95% CI | |  | OR | 95% CI | |
| **ZIP Code-Level** | |  |  |  |  |  |  |  |  |  |  |  |
|  | Median Household Income | **0.04** | **0.01** | **0.12** |  |  |  |  |  | **0.04** | **0.01** | **0.13** |
|  | GINI Coefficient | 1.03 | 0.62 | 1.71 |  |  |  |  |  | 1.27 | 0.77 | 2.09 |
|  |  |  |  |  |  |  |  |  |  |  |  |  |
| **State-Level** | |  |  |  |  |  |  |  |  |  |  |  |
|  | Median Household Income |  |  |  |  | 0.88 | 0.67 | 1.17 |  | 1.74 | 0.87 | 3.47 |
|  | GINI Coefficient |  |  |  |  | 0.87 | 0.59 | 1.28 |  | 0.53 | 0.23 | 1.23 |
| * Bolded values are statistically significant at an alpha of 0.05 | | | | | | | | | | | | |
| ^1^ Cases and controls were selected from the 34 states and four counties in California participating in the CDC's National Violent Death Reporting System (NVDRS). Case units were defined as the 250 ZIP codes with the highest per capita incidence of violent homicide deaths in 2017. Selected cases had $\geq$ 5 deaths. ZIP codes eligible for selection as control units (i) had no violent deaths in 2017 and (ii) were located within the 35 NVDRS states. Eligible controls had a population $\geq$ 25,000 and were randomly selected with replacement. | | | | | | | | | | | | |
| Model 1 adjusted for zip-code level variables | | | |  |  |  |  |  |  |  |  |  |
| Model 2 adjusted for state-level variables | | |  |  |  |  |  |  |  |  |  |  |
| Model 3 adjusted for all variables | |  |  |  |  |  |  |  |  |  |  |  |
